# Cobalt-Porphyrin-Platinum-Functionalized Reduced Graphene Oxide Hybrid Nanostructures: A Novel Peroxidase Mimetic System For Improved Electrochemical Immunoassay

**DOI:** 10.1038/srep15113

**Published:** 2015-10-13

**Authors:** Jian Shu, Zhenli Qiu, Qiaohua Wei, Junyang Zhuang, Dianping Tang

**Affiliations:** 1 Key Laboratory of Analysis and Detection for Food Safety (Ministry of Education & Fujian Province), Institute of Nanomedicine and Nanobiosensing, Department of Chemistry, Fuzhou University, Fuzhou 350108, P.R. China

## Abstract

5,10,15,20-Tetraphenyl-21H,23H-porphine cobalt flat stacking on the reduced graphene oxide with platinum nanoparticles (PtNPs/CoTPP/rGO) were first synthesized and functionalized with monoclonal rabbit anti-aflatoxin B_1_ antibody (anti-AFB_1_) for highly efficient electrochemical immunoassay of aflatoxin B_1_ (AFB_1_) in this work. Transmission electron microscopy (TEM), atomic force microscope (AFM) and spectral techniques were employed to characterize the PtNPs/CoTPP/rGO hybrids. Using anti-AFB_1_-conjugated PtNPs/CoTPP/rGO as the signal-transduction tag, a novel non-enzymatic electrochemical immunosensing system was designed for detection of target AFB_1_ on the AFB_1_-bovine serum albumin-functionalized sensing interface. Experimental results revealed that the designed immunoassay could exhibit good electrochemical responses for target analyte and allowed the detection of AFB_1_ at a concentration as low as 5.0 pg mL^−1^ (5.0 ppt). Intra- and inter-assay coefficients of variation were below 10%. Importantly, the methodology was further validated for analyzing naturally contaminated or spiked blank peanut samples with consistent results obtained by AFB_1_ ELISA kit, thus providing a promising approach for quantitative monitoring of organic pollutants.

Detectable signal amplification and noise reduction are one of the most important strategies in lowering the detection limit and increasing the sensitivity of the assay method, particularly immunoassay development^1^,^2^. Typically, natural enzymes including horseradish peroxidase and alkaline phosphatase have shown great application potentials because of their unique advantages: high catalytic activity, high specificity and easy conjugation with proteins^3^,^4^,^5^. Despite these extensive developments, natural enzymes often suffer from inherent shortcomings such as high cost of preparation and purification, low operational stability, sensitivity of catalytic activity to environmental conditions and difficulties in recovering^6^. An alternative approach that combines with high-efficiency biomimetic catalysts would be advantageous.

Porphyrins (a group of heterocyclic macrocycle organic compounds) are composed of four modified pyrrole subunits interconnected at their alpha carbon atoms *via* methane bridges^7^,^8^,^9^,^10^. Compared with natural enzymes, metalloporphyrins are low molecular weight and show superior thermal stability along with higher pH tolerance^11^,^12^,^13^,^14^,^15^,^16^. Nevertheless, direct application of metalloporphyrins in aqueous solution is usually challenging owing to the formation of catalytically inactive dimmers in the oxidizing reaction media^17^. More unfavorably, the biomolecules such as antibodies and aptamers are difficultly conjugated to the metalloporphyrins, which limits their practical applications. To address these problems, various methods have been developed to heterogenize metalloporphyrin catalysts including covalent bond formation, ion-pair formation, encapsulation or immobilization on the nanomaterials^18^,^19^,^20^.

As an alternative solution, graphene oxide (GO) has been demonstrated as the promising candidate to heterogenize metalloporphyrins^21^. One major advantage of using nanostructures is that one can control and tailor their properties in a very predictable manner to meet the needs of specific applications since nanomaterials can provide unique chemical and physical properties (in comparison with bulk materials) enabling new and advanced functions. It has been proven that porphyrin derivatives could be loaded onto the two accessible surfaces of graphene oxide *via* hydrophobic interactions and π-π stacking^22^. Despite many advances in this field, there is still the request for exploring innovative, highly efficient and stable biomimetic catalysts to improve the sensitivity and simplicity of the immunoassays.

Owing to the well-defined structures, hybrid nanostructures can broaden significantly to encompass a large variety of systems made of distinctly dissimilar components and mixed at the nanometer scale^23^. Recent research has demonstrated that the combination of graphene with nanomaterials such as nanoparticles, thereby forming graphene-nanoparticle hybrid structures, offers a number of additional unique physicochemical properties and functions that are both highly desirable and markedly advantageous for biological applications when compared to the use of either material alone^24^. Metallic platinum is one of the most attractive noble metal catalysts due to their outstanding catalytic ability. For example, the Qin group has demonstrated that platinum nanoparticles were much more active and stable toward the catalytic decomposition of hydrogen peroxide (H_2_O_2_) than catalase (one kind of bio-enzyme)^25^. Zhao *et al.* reported an advanced electrocatalyst with exceptional electrocatalytic activity *via* ultrafine platinum-based trimetallic nanoparticles on pristine graphene^26^. The noble-metal nanostructures hybridized with graphene possess high catalytic activity and rapidly transfer the electrons acquired from the catalytic process of the noble metal to substrate because the bond energy between platinum and CO_ads_ could be decreased due to the electron transfer from nickel to platinum via the reduction of DOS at the Fermi level during methanol electro-oxidation^26^,^27^. Inspired by these advantages, our motivation in this work is to combine the merits of platinum-graphene hybrid nanostructures with metalloporphyrins for the development of highly efficient electrochemical immunoassay.

Aflatoxins are highly toxic secondary metabolites produced by a number of different fungi, and can be present in a wide range of food and feed commodities^28^. They are potent toxic, carcinogenic, mutagenic, and immunosuppressive agents. The major aflatoxins of concern are designated as B_1_, B_2_, G_1_, and G_2_, however, aflatoxin B_1_ (AFB_1_) is usually predominant and most hazardous^29^. Thus, there is a need for development of validated analytical methods for rapid and cost effective screening of aflatoxins on a large scale and at low concentration levels. Herein, we introduce a novel peroxidase mimetic system for sensitive electrochemical detection of AFB_1_, as a model analyte, based on cobalt-porphyrin-platinum-functionalized reduced graphene oxide (Fig. 1). First, 5,10,15,20-tetraphenyl-21H,23H-porphine cobalt (CoTPP) flat stacking on the reduced graphene oxide (rGO) with platinum nanoparticles (PtNPs) is synthesized by using a wet-chemistry method. Then the as-synthesized PtNPs/CoTPP/rGO is utilized as the recognition element for the labelling of monoclonal rabbit anti-AFB_1_ antibody (anti-AFB_1_). Using the PtNPs/CoTPP/rGO as biomimetic catalyst and H_2_O_2_ as the substrate, a competitive-type electrochemical immunoassay is developed for the detection of AFB_1_ on AFB_1_-bovine serum albumin (AFB_1_-BSA)-functionalized sensing interface. The detectable signal derives from the biomimetic catalyst toward the catalytic reduction of H_2_O_2_.

## Results

As is well known, porphyrins can be conjugated to single-layer graphene nanosheets with SP^2^ hybrid orbital to form the organic-inorganic hybrid nanocomposites on the basis of the π-π stacking interaction^30^. Figure 2A shows typical UV-vis absorption spectroscopes of GO after interaction with CoTPP with different times under the ultrasonic oscillation. At the first, the spectrum of the mixture containing CoTPP and GO featured an intense Soret band at 416 nm and two Q bands at 546 nm and 586 nm, which was ascribed to the characteristic absorption of CoTPP (Fig. 3G, inset). When CoTPP was interacted with GO under ultrasonic oscillation, a new characteristic peak was observed at 432 nm. Moreover, the absorbance at 432 nm gradually increased with the increasing time of ultrasonic oscillation, while the absorbance at 416 nm gradually decreased, which was in accordance with the previous report^31^. The reason for the red shift of the absorption peak could be explained by electron transfer and molecular flattening mechanism^30^. The stacking interaction of two π electron systems between CoTPP and GO caused the bathochromic shift^30^,^32^. During the preparation precess, we also observed that CoTPP was difficultly assembled to GO within a relatively short incubation time in the absence of ultrasonic oscillation, which was different from rGO^31^. By increasing the amount of GO and prolonging the assembly time between GO and CoTPP, however, CoTPP could be still immobilized onto the GO without ultrasonic oscillation, as seen from Fig. 2B. We speculated, the reason might be most likely as the consequence of the fact that the ultrasonic oscillation decreases the resistance of the functional groups on the GO and prompts CoTPP to flatten on GO. The results revealed that the ultrasonic oscillation accelerated the assembly of CoTPP on the GO.

To further demonstrate that CoTPP could be assembled onto the GO, we used fluorescence spectrum to investigate CoTPP before and after interaction with different-concentration GO since GO can show well fluorescence quenching effect toward many fluorescent substrates (Fig. 2C). Pure CoTPP displayed a strong fluorescent peak at 655 nm and a weak fluorescent peak at 718 nm in the DMF-ethanol mixture. The fluorescent intensity of CoTPP gradually decreased with the increasing GO. The shape of absorbance of CoTPP changed significantly while emission peak showed any changes except intensity, which indicated free and adsorbed CoTPP coexisted. Accordingly, the static/dynamic quenching coexisted and related constant were determined from the following Stern-Volmer equation^33^:


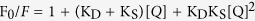


Where, F_0_ and F denote the fluorescence intensities of the fluorescent substance in the absence and in the presence of quencher concentration of [Q]. K_D_ and K_S_ are dynamic quenching constant and static quenching constant, respectively. A straight line fitted by using [F_0_/F–1]/[Q] versus [Q] (Fig. 2D). The K_D_ and K_S_ values were calculated by a quadratic equation about the intercept (K_D_ + K_S_) and slope (K_D_K_S_). The results of the calculation was K_D_ = 0.0379, K_S_ = 3.4043. The K_S_ is nearly 100 times as big as K_D_. This demonstrated static quenching that non-fluorescent complex formation was the main reason for decreasing fluorescence intensity in this concentration range. Quenching mechanism may be attributed to the excited state electron of CoTPP transfer to GO through the π-π interaction. Therefore, the CoTPP could be assembled onto the GO. We also noticed that fluorescence would complete quenching with GO risen continuously but the straight line bend to Y axis. This result showed dynamic quenching existed and gradually played major role on the processes of fluorescence quencher.

Another two concerns arise as to whether (i) PtNPs could be attached to the CoTPP/GO, and (ii) GO on the CoTPP/GO could be reduced to the rGO. Although the strong sonication was employed during the preparation process and the large-sized GO was removed by centrifugation, the little residue of large sheets could not be avoided^34^. As seen from atomic force microscope (AFM, Bruker Nano Inc, USA) image in Fig. 3A, the size of the synthesized GO was 120–180 nm with a clear thickness of ~1.0 nm. The single or very thin layers with the sizes well suited for loading nanoparticles, biomolecular and drug, and have been widely used in the immunoassays and drug delivery systems^35^,^36^,^37^. The transmission electron microscope (TEM, Model H-7650, Hitachi Instruments, Japan) image of GO exhibits the typical wrinkle morphology (Fig. 3B). Compared with the GO alone, many nanoparticles could be observed in the TEM image of PtNPs/CoTPP/rGO (Fig. 3C). Such a sheet-shaped structure provided a large surface area for the assembly of the nanoparticles. Furthermore, the successful assembly of PtNPs and GO reduction were also verified by X-ray photoelectron spectroscopy (XPS, ESCALAB 250, Thermo Scientific, USA). As shown in Fig. 3D, the spectrum of CoTPP/GO exhibited not only C_1s_ and O_1s_ peaks, but also displayed a distinct N_1s_ peak in comparison with pure GO. Appearance of N_1s_ peak suggested the existence of CoTPP. In contrast, a new peak at 72.6 eV (Pt_4f_) was appeared for PtNPs/CoTPP/rGO, indicating that platinum element was assembled to the CoTPP/rGO. In addition, a decline in the relative contents of elements O was observed at the hybrid nanostructure, suggesting that the oxygen functional groups on the surface of GO was reduced by ascorbic acid (AA). Significantly, introduction of CoTPP and PtNPs on the rGO could largely improve the dispersity of the rGO in the water (Fig. 3E, left). In contrast, the rGO alone without any modification was aggregated together after being stored for three months at room temperature (Fig. 3E, right). The stability of PtNPs/CoTPP/rGO in aqueous solution through electrostatic repulsion was characterized by zeta potential. A negative zeta potential value of −50.2 mV was found for PtNPs/CoTPP/rGO, which was higher than that of GO (−48.7 mV). The long-term stability was favorable for the development of the subsequent electrochemical immunoassay.

The Fourier transform infrared (FTIR) spectroscopy (Fig. 3F) and UV-vis absorption spectra (Fig. 3G) were also used to confirm the above-mentioned results. FTIR spectroscopy of CoTPP/GO exhibits typical peaks for both GO and CoTPP with slight shift. The peaks at 808 cm^−1^ and 748 cm^−1^ correspond to the skeletal vibration of porphyrin ring. The C = N stretching vibration of CoTPP at 1630 cm^−1^ shifted to 1670 cm^−1^ when CoTPP assemble onto GO. The peak at 1045 cm^−1^, 1220 cm^−1^ and 1730 cm^−1^ corresponds to functional groups C-O and C = O stretching vibrations of GO. After CoTPP/GO was reacted with H_2_PtCl_6_ and AA, FTIR spectroscopy of PtNPs/CoTPP/rGO revealed little change of typical peaks of CoTPP, while the characteristic peaks at 1730 cm^−1^ and 1220 cm^−1^ of GO disappeared, which suggesting functional groups of GO were almost reduced. On the other hand, from the UV-vis absorption spectra, we can clearly observe the change of characteristic absorbance. GO and rGO show their own special absorbance at 229 nm and 266 nm, respectively. Besides an intense absorbance of GO at 229 nm, when CoTPP was assemble onto the GO, CoTPP/rGO exhibits a week absorbance at 432 nm. After CoTPP/GO was reacted with H_2_PtCl_6_ and AA, PtNPs/CoTPP/rGO also exhibits a week absorbance at 432 nm and another intense absorbance of rGO at 266 nm. This demonstrates only H_2_PtCl_6_ and GO would be reduced and CoTPP would not be affect in this reaction. The change of FTIR and UV-vis spectra confirmed the assembly of CoTPP and reduction of GO.

In addition, Raman spectroscopy was also employed as powerful demonstration that GO was reduced during the synthesis because Raman spectroscopy is a powerful tool that can provide the important information on crystallite size, stereochemical purity and the sp^2^–sp^3^ hybridization, *e.g.*, the sp^2^ structural changes of GO, CoTPP/GO and PtNPs/CoTPP/rGO^38^. As shown in Fig. 3H, three nanostructures displayed a strong D peak and a strong G peak with a distinguishable ratio in the intensity (I_D_/I_G_). The G band was intrinsic Raman modes of the graphite material that assigned to the E_2g_ vibrational mode of Brillouin zone, while the D band represented the k-point phonon model of the A_1g_^39^. When CoTPP was assembled on GO, I_D_/I_G_ decreased from 0.92 (curve ‘*a*’) to 0.86 (curve ‘*b*’), indicating that the big π structure on the surface of GO was formed. Thus, CoTPP could be assembled on GO through π-π stacking interaction. When CoTPP/GO hybrid nanostructures were reacted with H_2_PtCl_6_ and AA, however, the I_D_/I_G_ of the nanostructures increased to 1.17 (curve ‘*c*’), which was ascribed to the cleavage of carbon-carbon bond during the reduction process of GO, thus decreasing the relative size (*L*a) and order degree of graphene^40^,^41^.

For successful development of PtNPs/CoTPP/rGO-based non-enzymatic electrochemical immunoassay, a pivotal point is whether the as-prepared PtNPs/CoTPP/rGO could efficiently catalyze the reduction of hydrogen peroxide. To clarify this issue, several control tests were carried out under the same experimental conditions by using 3,3′,5,5′-tetramethylbenzidine (TMB) and H_2_O_2_ as the substrates. The assay was recorded and registered by using the colorimetric detection and UV-vis absorption spectroscopy (Fig. 4A,B) (*Note*: 10 *μ*L of 1 mg mL^−1^ nanostructures, 50 *μ*L of 1.0 mM TMB and 50 *μ*L of 0.1 mM H_2_O_2_ used in this case in pH 7.0 PBS). As shown from curve ‘*a*’ in Fig. 4A, in a short time, almost no absorbance was observed toward the mixture containing GO, TMB and H_2_O_2_, indicating that GO lack remarkable catalytic capability to H_2_O_2_. However, the absorbance increased when using CoTPP/GO (curve ‘*b*’) or PtNPs/rGO (curve ‘*c*’) as the peroxidase mimics, respectively. Significantly, when PtNPs/CoTPP/rGO was reacted with TMB/H_2_O_2_, a strong absorbance was acquired (column ‘*d*’). For comparison, we also investigated the absorbance of the synthesized PtNPs/CoTPP/rGO after reaction with TMB or H_2_O_2_ alone, respectively. As shown from column ‘*e-f*’, no absorbance was obtained. These results were in accordance with the colorimetric colors in Fig. 4B. Therefore, we could make a conclusion that the as-synthesized PtNPs/CoTPP/rGO nanostructures could be used as the peroxidase-like mimics to catalyze the reduction of H_2_O_2_.

To further clarify the high catalytic efficiency of the as-prepared PtNPs/CoTPP/rGO toward H_2_O_2_ reduction, four nanostructures including PtNPs/CoTPP/rGO, PtNPs/rGO, CoTPP/GO and GO were directly dropped onto the cleaned GCE, respectively, which were monitored in pH 7.0 PBS by using differential pulse voltammetry (DPV) in the absence and presence of H_2_O_2_ (Fig. 4C). Almost no peak currents were observed at GO-modified GCE, regardless of without and with the H_2_O_2_ (curve ‘*a*_0_’ versus curve ‘*a*’). However, a relatively weak catalytic current could be acquired when using PtNPs/rGO or CoTPP/GO-modified GCE in the absence of H_2_O_2_ (curve ‘*b*’ and curve ‘*d*’). Inspiringly, a large shift in the DPV peak current was achieved when using PtNPs/CoTPP/rGO as the substrates (curve ‘*c*_0_’ versus curve ‘*c*’). The results revealed that the as-prepared PtNPs/CoTPP/rGO could catalyze the reduction of H_2_O_2_ with high efficiency.

To achieve a high-sensitivity PtNPs/CoTPP/rGO-based non-enzymatic electrochemical immunoassay, some experimental parameters including the antigen-antibody reaction and pH of detection solution would be investigated. Figure 5A represents the effect of pH of PBS on the DPV peak current of non-enzymatic electrochemical immunoassay in the absence of target AFB_1_. The optimal current could be got at pH 7.0 PBS. A high or low pH would decrease the electrochemical signal. So, pH 7.0 PBS was utilized for the detection of target AFB_1_.

Another important parameter is the incubation time between antigen and antibody. Usually, it takes some time for the antigen-antibody reaction. A short incubation time is unfavorable for the detection of target analyte, especial at a relatively low concentration. In this case, the judgment was based on the reaction between AFB_1_-BSA-AuNPs-GCE and anti-AFB_1_- PtNPs/CoTPP/rGO in the absence of target AFB_1_. As seen from Fig. 5B, the electrochemical signal increased with the increment of incubation time, and then reached a steady-state current after 25 min. A long incubation time did not obviously change the detectable signal. Thus, 25 min was employed for the antigen-antibody reaction.

Under the optimal conditions, the as-prepared AFB_1_-BSA-AuNPs-GCE and anti-AFB_1_- PtNPs/CoTPP/rGO were used for the monitoring of target AFB_1_ by using differential pulse voltammetry in pH 7.0 PBS containing 50 *μ*M H_2_O_2_ with a competitive-type immunoassay mode. As shown from Fig. 6A, the currents decreased with the increasing AFB_1_ concentrations in the sample. A linear dependence between the change of DPV peak current (*μ*A) (relative to background signal) and the logarithm of AFB_1_ level (ng mL^−1^) in the sample could be acquired in the dynamic range from 0.005 to 5.0 ng mL^−1^ (ppb) with a detection limit (LOD) of 1.5 pg mL^−1^ (ppt) at a signal-to-noise ration of 3δ (where δ is the standard deviation of a blank solution) (inset, Fig. 6A). The regression equation could be fitted to *y* (*μ*A) = 9.62 × Ln *C*_[AFB1]_ + 51.001 (ng mL^−1^, *R*^2^ = 0.9987). Apparently, the LOD of PtNPs/CoTPP/rGO-based electrochemical immunoassay toward AFB_1_ was comparable with commercialized available AFB_1_ ELISA kits obtained from different companies (*e.g.* Quicking Biotech: 100 ppt; MaxSignal®: 50 ppt; MyBioSource: 250 ppt; Diagnostic Automation: 5 ppt), fluorescence polarization immunoassay (LOD: 1.0 ng mL^−1^)^42^, electrochemical impedance spectroscopy-based immunosensor (LOD: 30 pg mL^−1^)^43^, liquid chromatography-tandem mass spectrometry (LOD: 2.0 ng mL^−1^)^44^, immunogold-based optical detection (LOD: 7.0 pg mL^−1^)^45^, and SERS-based immunoassay (LOD: 0.1 ng mL^−1^)^46^. Due to the legal limit of AFB_1_ (<2.0 ng mL^−1^), the non-enzymatic electrochemical immunoassay could completely meet the requirement of AFB_1_ monitoring in foodstuffs.

Next, we determined the reproducibility and precision of non-enzymatic electrochemical immunoassay by using different batches of immunosensing probes for the analysis of three AFB_1_ samples with different levels including 10 pg mL^−1^, 0.1 ng mL^−1^ and 5.0 ng mL^−1^. Experimental results indicated that the relative standard deviations (RSD) by using the AFB_1_-BSA-AuNPs-GCE and anti-AFB_1_-PtNPs/CoTPP/rGO with the same batch were 6.7%, 8.2% and 5.8%, respectively, while those of the inter-assay with different batches were 9.3%, 8.9% and 9.7%, respectively. The results pointed out the importance of batch-wise preparation and use of AFB_1_-BSA-AuNPs-GCE and anti-AFB_1_-PtNPs/CoTPP/rGO.

Further, the specificity of non-enzymatic electrochemical immunoassay was monitored by using AFB_2_, AFG_1_, AFG_2_, and two protein antigens alpha-fetoprotein (AFP) and thyroid-stimulating hormone (TSH). As seen from Fig. 6B, AFG_1_, AFG_2_, AFP and TSH alone or in combination with the target did not cause the significant increase in the signal relative to the control test. However, the relatively strong signal toward AFB_2_ sample could be also easily explained by the fact that the used AFB_1_ antibody has a high cross-reactivity for AFB_2_, as described in our previous report^47^. Thus, the specificity of non-enzymatic electrochemical immunoassay was acceptable.

The feasibility of applying the non-enzymatic electrochemical immunoassay to assess aflatoxin levels in a complex matrix was also monitored. Initially, we spiked AFB_1_ standards with random concentrations into blank peanut homogenate, followed by a centrifugation and extraction procedure. Finally, these samples were measured by the non-enzymatic electrochemical immunoassay and the commercialized MaxSignal® AFB_1_ ELISA kit, respectively. Further, we also used the developed electrochemical immunoassay to analyze the naturally contaminated peanut samples by using the same method (*Note*: These samples purchased from the local supermarket were set for various times under dull and wet conditions to provoke growth of fungi and achieve a high-concentration of AFB_1_ prior to measurement). The aflatoxin content obtained by the non-enzymatic electrochemical immunoassay was calculated according to the above-mentioned regression equation (*y* = 9.62 × Ln *C*_[AFB1]_ + 51.001). The results are summarized in Table 1. Statistical comparison of the experimental results of the electrochemical immunosensors with ELISA was performed using a *t*-test for comparison of means preceded by the application of an F-test. The *t* statistics for each sample was calculated by using independent two-sample *t*-test with equal sample sizes and equal variance as follows:


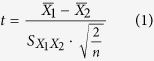


where


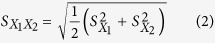


Here 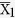 and 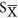 stand for the mean of each PRL sample estimated with the immunosensor and the standard error of the mean, respectively (1 = group one, 2 = group two), and *n* stands for the assay times for each sample. No significant differences were encountered between the two methods at the 0.05 significance level (Table 1) because the *t*_exp_ were in all cases below than *t*_crit_ (*t*_crit[4,0.05]_ = 2.77). Therefore, the developed non-enzymatic electrochemical immunoassay could be utilized for quantitative monitoring of target AFB_1_ in real samples.

## Discussion

To develop a high-efficiency non-enzymatic immunosensing protocol, signal amplification and noise decrease are very crucial during the electrochemical measurement. In this work, the signal is amplified by the organic-inorganic PtNPs/CoTPP/rGO hybrid nanostructures toward the catalytic reduction of H_2_O_2_. Target AFB_1_ is monitored by using PtNPs/CoTPP/rGO-labeled anti-AFB_1_ antibody as the signal-transduction tag. The low background signal derives from the non-electroactive AFB_1_-BSA-modified AuNPs/GCE. CoTPP molecules are assembled onto GO through the hydrophobic interactions and π-π stacking, whereas PtNPs are formed by *in situ* chemical reduction. AFB_1_-BSA conjugates are immobilized onto the nanogold-functionalized GCE because of the interaction between cysteine or NH_3_^+^-lysine residue of protein and gold element. In the presence of target AFB_1_, the analyte is competed with the immobilized AFB_1_-BSA on the GCE for the labeled anti-AFB_1_ antibody on the PtNPs/CoTPP/rGO, thereby resulting in the decreasing catalytic current. Experimental results revealed that introduction of PtNPs/CoTPP/rGO could display a wide linear range and a relatively low detection limit for the determination of target AFB_1_.

In conclusion, we demonstrated the development of a simple and feasible non-enzymatic electrochemical immunoassay with sensitivity enhancement for the detection of aflatoxins (AFB_1_ used as a model analyte) in foodstuff by coupling with a novel peroxidase mimetic system, PtNPs and CoTPP-functionalized rGO. rGO with a large volume-to-surface ratio was designed as the affinity support for the assembly of CoTPP and PtNPs, and CoTPP and PtNPs were utilized as the peroxidase mimics for the catalytic reduction of hydrogen peroxide. Compared with natural enzyme-labeled strategies, nanocatalyst-based assay system is not susceptible to interference and the detection conditions during the signal generation stage, *e.g.* pH, temperature and instability cause by structural unfolding. Importantly, the system was capable continuously carrying out all steps within <30 min for one sample including incubation, washing and measurement, which was largely less than that of the commercialized AFB_1_ ELISA kits (~3 h). Moreover, PtNPs/CoTPP/rGO-based non-enzymatic immunoassay does not require sophisticated instruments and is well suitable for high-throughput biomedical sensing and application for other areas. Nevertheless, only one disadvantage of using PtNPs/CoTTP/rGO as the electrochemical signal-generation tag is unsuitable for the detection of light-deactivated pathogens or biomarkers. Although the methodology mainly focused on target AFB_1_, it is easily extended to determine other small molecular biotoxins or low-abundant protein by controlling the corresponding antigen or antibody.

## Methods

### Materials and reagents

Monoclonal rabbit anti-AFB_1_ antibody and AFB_1_-BSA conjugate were gifted from Oil Crops Research Institute of the Chinese Academy of Agricultural Sciences (Wuhan, China). AFB_1_, AFB_2_, AFG_1_ and AFG_2_ standards (in acetonitrile) with various concentrations were purchased from Express Technol. Co., Ltd. (Beijing, China). BSA, ascorbic acid, and Co(CH_3_COO)_2_ 4H_2_O, H_2_PtCl_6_·6H_2_O and HAuCl_4_·4H_2_O were achieved from Sinopharm Chem. Re. Co., Ltd. (Shanghai, China). 5,10,15,20-Tetraphenyl-21H,23H-porphine cobalt (CoTPP) was purchased from Luminescence Technol. Corp. (Taiwan, China). All other reagents were of analytical grade and were used without further purification. Ultrapure water obtained from a Millipore water purification system (18.2 MΩ cm^−1^, Milli-Q, Merck Millipore, Darmstadt, Germany) was used in all runs. Phosphate-buffer saline (PBS) and Tris-HCl buffer were obtained from Sigma-Aldrich.

### Food samples and extraction procedure

Peanut matrix for further addition calibration was prepared as follows: 5.0 g of ground blank peanut sample was milled first with a small Midea Food Mixer (BP252AG, Guangdong, China) and then extracted with MeOH/water (37.5 mL, 80:20, v/v) under gentle stirring for 60 min followed by filtration. 20 mL of the extract was diluted into 60 mL of water (20%, v/v, final content of MeOH). Finally, calibration solutions were prepared by spiking aliquots of an AFB_1_ stock solution (acetonitrile as solvent) into different volumes of diluted extract. Contaminated peanut samples were made available as slurry (150% of water, v/w). A portion of 7.5 g of slurry was extracted with 18 mL of MeOH for 1 h followed by filtration. To 5.0 mL of extract, 15.0 mL of pH 7.4 PBS was added to obtain a final concentration of 20% (v/v) MeOH.

### Preparation and bioconjugation of PtNPs/CoTPP/rGO with anti-AFB1 antibody

Prior to experiments, GO was prepared from graphite powder by typical Hummer’s method. Following that, the as-prepared GO was used for the assembly of CoTPP on the basis of a simple self-assembly process. Briefly, 5 mg of GO was initially thrown into 2 mL ultrapure water, and the resultant mixture was vigorously sonicated for 60 min at room temperature to obtain a homogeneous suspension. Afterwards, 0.1 mmol of CoTPP, dispersed in 1.0 mL DMF/water (4:1, v/v), was dropped into the suspension. The resulting mixture was further sonicated for 60 min. During this process, CoTPP was assembled onto the graphene oxide by the hydrophobic interactions and π-π stacking. The cobalt-porphyrin-functionalized graphene oxide (designated as CoTPP/GO) was obtained through centrifugation (10,000 *g*, 10 min), and re-dispersed into 2 mL ultrapure water.

Next, the obtained CoTPP/GO was utilized for the assembly of PtNPs according to the literature with minor modification^48^. 10 *μ*L of H_2_PtCl_6_·6H_2_O (23.4 mM) was initially dropped into the above-prepared CoTPP/GO suspension, and the mixture was stirred for 12 h at room temperature. Following that, 500 *μ*L of ascorbic acid (5.0 mM) was added into the mixture. During the procedure, the Pt (IV) was reduced to PtNPs and GO was reduced to the rGO. Finally, the cobalt-porphyrin-platinum-functionalized reduced graphene oxide (designated as PtNPs/CoTPP/rGO) were centrifuged, and re-dispersed in 2 mL ultrapure water for further use.

At the third step, the as-prepared PtNPs/CoTPP/rGO was employed for the bioconjugation of anti-AFB_1_ antibody based on the interaction between PtNPs and antibody. Initially, 100 *μ*L of 1.0 mg mL^−1^ anti-AFB_1_ antibody was added to the mixture and slightly stirred for 12 h at 4 °C. During this process, anti-AFB_1_ antibody was conjugated onto the surface of PtNPs through the formation of the Pt-S bond or Pt-NH bond between PtNPs and protein. Afterwards, the mixture was centrifuged for 20 min at 13,000 *g* at 4 °C, and the supernatant was removed. Subsequently, the as-prepared anti-AFB_1_-conjugated PtNPs/CoTPP/rGO (designated as anti-AFB_1_-PtNPs/CoTPP/rGO) was added into 2 mL PBS, pH 7.4, containing 0.05 wt% Tween-20, and stored at 4 °C when not in use.

### Preparation of AFB1-BSA-modified glassy carbon electrode

A thin layer of nanogold particles (AuNPs) was initially electrodeposited onto a cleaned glassy carbon electrode (GCE, 2 mm in diameter) (CHI Ins. Co. Ltd., Shanghai, China) by using a potential-step electrolysis with various pulse times (10, 30 and 60 s) in the mixture containing 0.5 M H_2_SO_4_ and 1.0 mM HAuCl_4_ (working range: +1.1–0 V). After washing twice with distilled water, the AuNPs-modified GCE was immersed into 0.1 mg mL^−1^ BSA-AFB_1_ solution, and reacted overnight at 4 °C. During the process, BSA-AFB_1_ conjugates were attached to the GCE *via* the interaction between cysteine/NH_3_^+^-lysine residues of BSA and AuNPs. Subsequently, the immunosensor (designated as AFB_1_-BSA-AuNPs-GCE) was incubated for 60 min at room temperature with 0.5 wt % BSA, and stored at 4 °C for use.

### Electrochemical measurement for target AFB1

**Figure 1:**
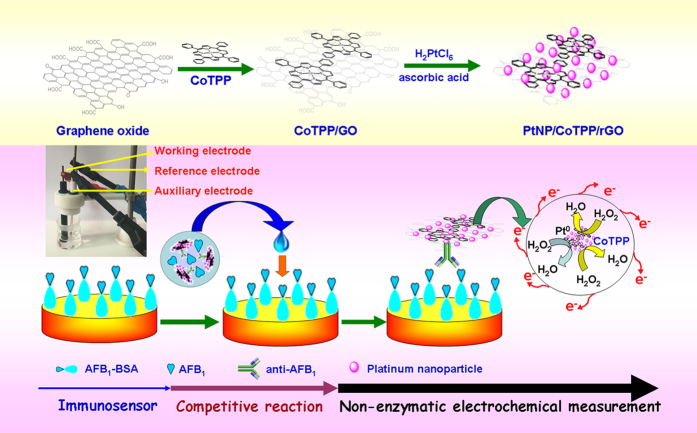
Schematic illustration of non-enzymatic competitive-type electrochemical immunoassay toward target AFB_1_ by using PtNPs/CoTPP/rGO as the signal-transduction tags (*Inserted photo*: Electrochemical measurement device).

**Figure 2:**
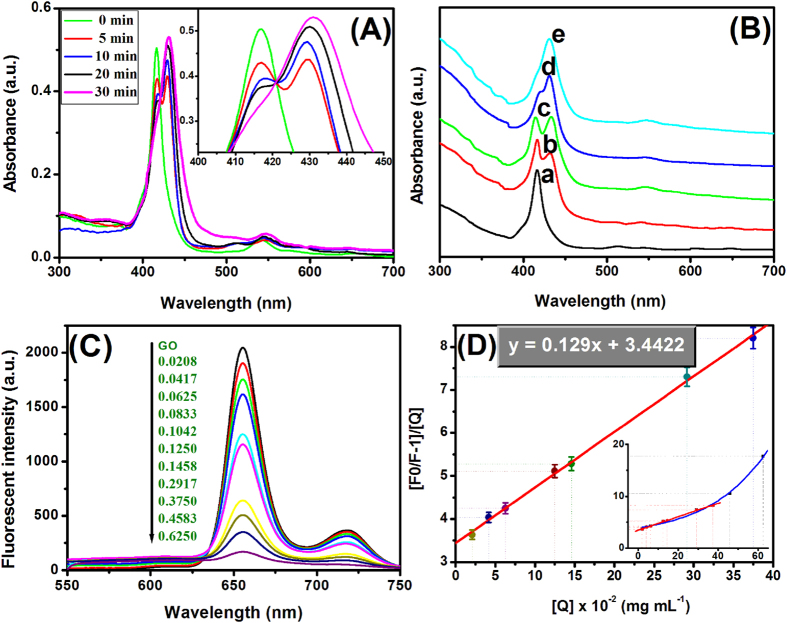
(**A**) UV-vis absorption spectra of 0.1 mmol CoTPP + 2.0 mg GO after interaction with different times under the ultrasonic oscillation (*Inset*: Magnification spectra at Soret band), (**B**) UV-vis absorption spectra of 0.1 mmol CoTPP + 10 mg GO after interaction with different times without ultrasonic oscillation, (**C**) fluorescent spectra of 0.1 mmol CoTPP after interaction with different-amount GO (mg), and (**D**) Stern-Volmer plots of fluorescence quenching (*Inset*: The plots of high-concentration GO).

**Figure 3:**
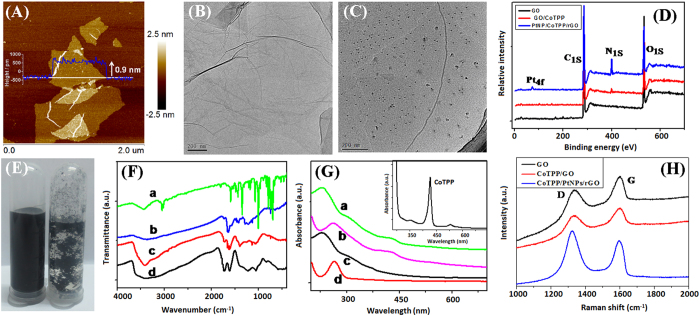
(**A**) AFM image of GO on the freshly cleaved mica; (**B**,**C**) typical TEM images of (**B**) GO and (**C**) PtNPs/CoTPP/rGO, (**C**) XPS spectra of GO, CoTPP/GO and PtNPs/CoTPP/rGO; (**E**) photographs of (left) PtNPs/CoTPP/rGO and (right) rGO after storing for three months at room temperature, respectively; (**F**) FTIR spectra of (**a**) CoTPP, (**b**) PtNPs/CoTPP/rGO, (**c**) CoTPP/GO and (**d**) GO; (**G**) UV-vis absorption spectra of (**a**) CoTPP/GO, (**b**) PtNPs/CoTPP/rGO, (**c**) GO and (**d**) rGO (*Inset*: the corresponding CoTPP); and (**H**) Raman spectra of GO, CoTPP/GO and PtNPs/CoTPP/rGO.

**Figure 4:**
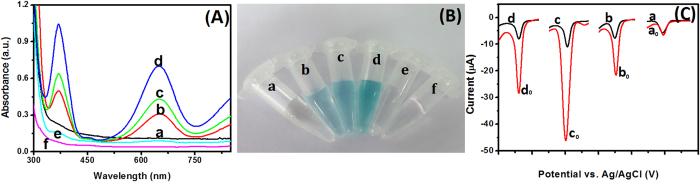
(**A**) Comparison of different components in the absorbance density: (**a**) GO + TMB + H_2_O_2_, (**b**) CoTPP/GO + TMB + H_2_O_2_, (**c**) PtNPs/rGO + TMB + H_2_O_2_, (**d**) PtNPs/CoTPP/rGO + TMB + H_2_O_2_, (**e**) PtNPs/CoTPP/rGO + TMB, and (**f**) PtNPs/CoTPP/rGO + H_2_O_2_; (**B**) the corresponding photographs in figure (**A**); and (**C**) DPV curves of (**a**_**0**_,**a**) GO-modified GCE, (**b**_**0**_,**b**) CoTPP/GO-modified GCE, (**c**_**0**_,**c**) PtNPs/CoTPP/rGO-modified GCE and (**d**_**0**_,**d**) PtNPs/rGO-modified in pH 7.0 PBS in the (**a**,**b**,**c**,**d**) absence and (**a**_**0**_,**b**_**0**_,**c**_**0**_,**d**_**0**_) presence of H_2_O_2_.

**Figure 5:**
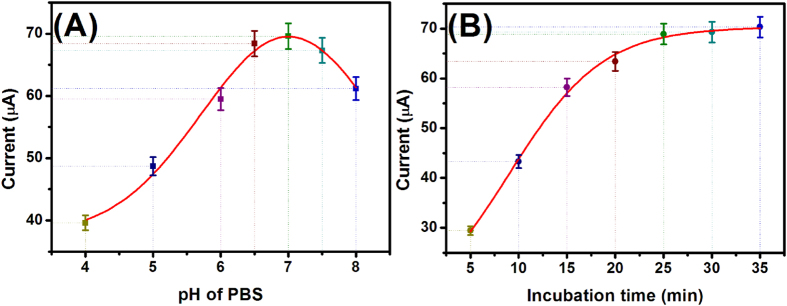
The effects of (**A**) pH of PBS and (**B**) incubation time for the antigen-antibody reaction on DPV peak current of PtNPs/CoTPP/rGO-based non-enzymatic electrochemical immunoassay in the absence of target AFB_1_. Each data point represents the average value obtained from three different measurements.

**Figure 6:**
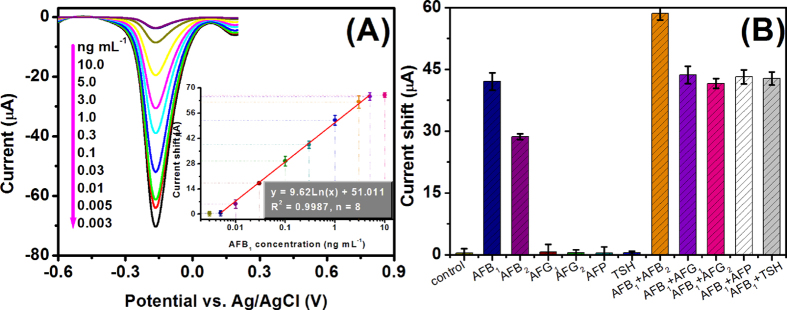
(**A**) DPV response curves of PtNPs/CoTPP/rGO-based non-enzymatic electrochemical immunoassay toward different-concentration AFB_1_ standards in pH 7.0 PBS containing 50 *μ*M H_2_O_2_ (*Inset*: Calibration plots), and (**B**) the specificity of the developed immunoassay against target AFB_1_ (0.1 ng mL^−1^), AFB_2_ (0.1 ng mL^−1^), AFG_1_ (0.1 ng mL^−1^), AFG_2_ (0.1 ng mL^−1^), AFP (50 ng mL^−1^) and TSH (50 ng mL^−1^). Each data point represents the average value obtained from three different measurements.

Figure 1 represents the measurement process of non-enzymatic electrochemical immunoassay toward target AFB_1_ with a competitive-type assay format by using the as-prepared PtNPs/CoTPP/rGO as a novel peroxidase mimetic system on AFB_1_-BSA-modified electrode. Electrochemical measurement was carried out on an AutoLab *μ*AUTIII electrochemical workstation (Eco Chemie, The Netherlands) with a modified GCE working electrode, a Pt wire-auxiliary electrode and an Ag/AgCl reference electrode. The assay was carried out as follows: (i) 10 *μ*L of the mixture containing 5-*μ*L AFB_1_ samples or standards with different concentrations and 5 *μ*L of the above-prepared anti-AFB_1_-PtNPs/CoTPP/rGO was dropped onto the AFB_1_-BSA-modified electrode and incubated for 25 min at 37 °C to form the antigen-antibody immunocomplex; and (ii) the resultant immunosensor was placed into pH 7.0 PBS containing 50 *μ*M H_2_O_2_ and differential pulse voltammogram (DPV) was monitored from +200 mV to −600 mV (Amplitude: 50 mV; width: 50 ms) (*Note*: The resulting immunosensor was washed with distilled water after incubation, and the immunosensor was sealed with a centrifugal tube during the incubation). A baseline correction of the resultant voltammogram was performed with workstation software. The peak current was collected and registered as the sensor signal relative to target AFB_1_ concentration. All measurements were performed at room temperature (25 ± 1.0 °C).

### Safety

Aflatoxins are powerful hepatotoxins and carcinogens, so great care should be taken to avoid personal exposure and potential laboratory contamination. All items coming in contact with aflatoxins (glassware, vials, tubes, etc.) were immersed in a 10% bleach solution for 1–2 h before they were discarded. Pure aflatoxin standard was handled in a hood with extreme caution.

## Additional Information

**How to cite this article**: Shu, J. *et al.* Cobalt-Porphyrin-Platinum-Functionalized Reduced Graphene Oxide Hybrid Nanostructures: A Novel Peroxidase Mimetic System for Improved Electrochemical Immunoassay. *Sci. Rep.* **5**, 15113; doi: 10.1038/srep15113 (2015).

## Figures and Tables

**Table 1:** Results obtained by the non-enzymatic electrochemical immunoassay and commercial AFB_1_ ELISA kit (MaxSignal AFB_1_ ELISA) for spiked or naturally contaminated peanut samples.

Type	No.	Method; concentration (ng mL^–1^, n = 3)^a^		
		Electrochemical immunoassay	AFB_1_ ELISA kit	*t*_exp_
spiked peanut	1	0.008 ± 0.001	No detection	No application
	2	0.54 ± 0.12	0.51 ± 0.32	0.14
	3	1.32 ± 0.21	1.39 ± 0.21	0.41
	4	4.67 ± 0.89	4.51 ± 0.43	0.28
	5	9.32 ± 0.76	9.65 ± 0.89	0.49
naturally contaminated peanut	1	4.32 ± 0.88	4.12 ± 1.01	0.26
	2	9.54 ± 1.32	9.76 ± 1.13	0.22
	3	6.45 ± 1.12	6.23 ± 0.89	0.26
	4	3.21 ± 0.89	3.11 ± 0.78	0.15
	5	10.3 ± 1.73	10.9 ± 1.32	0.45

^a^Each sample was determined in triplicate, and the high-concentration AFB_1_ sample was assayed with an appropriate dilution.
